# Supplementary material for: Injury incidence, characteristics and burden among female sub-elite futsal players: a prospective study with three-year follow-up
Source: PeerJ. 2019 Nov 5;7:e7989. doi: 10.7717/peerj.7989 (PMC6839517; doi:10.7717/peerj.7989)
Supplement: Supplemental Information 1 [file peerj-07-7989-s001.docx]

| **Appendix A1**. Definitions used to include studies. | |
| --- | --- |
| **Term** | **Definition** |
| Injury | Any physical complaint sustained by a player that results from a futsal match or futsal training, irrespective of the need for medical attention or time loss from futsal activities. |
| Time loss injury | Injury that results in a player being unable to take a full part in future futsal training or match play. |
| Recurrent injury | An injury of the same type and at the same site as an index injury and which occurs after a player’s return to full participation from the index injury. |
| Injury severity | The number of days that have elapsed from the date of injury to the date of the player’s return to full participation in team training and availability for match selection. Injuries are grouped as:  *Slight / Minimal* Absence (1-3 days)  *Minor / Mild* Absence (4-7 days)  *Moderate* Absence (8-28 days)  *Major / Severe* Absence (>28 days) |
| Match exposure | Play between teams from different clubs. |
| Training exposure | Team-based and individual physical activities under the control or guidance of the team’s coaching or fitness staff that are aimed at maintaining or improving players’ futsal skills or physical condition. |
| Overuse injury | An injury caused by repeated microtrauma without a single, identifiable event responsible for the injury. |
| Traumatic injury | Injury with sudden onset and known cause. |
| Injury location | - Head and neck (Head/face; Neck/cervical spine) - Upper limbs (Shoulder/clavicula; Upper arm; Elbow; Forearm; Wrist; Hand/finger/thumb) - Trunk (Sternum/ribs/upper back; Abdomen; Lower back/pelvis/sacrum) - Lower limbs (Hip/groin; Thigh; Knee; Lower leg/Achilles tendon; Ankle; Foot/toe) |
| Type of injury grouping | - Fractures and bone stress - Joint (non-bone) and ligament [Dislocation/subluxation; Sprain/ligament injury; Lesion of meniscus or cartilage] - Muscle and tendon [Muscle rupture/tear/strain/cramps; Tendon injury/rupture/tendinosis/bursitis] - Contusions [Haematoma/contusion/bruise] - Laceration and skin lesion [Abrasion; Laceration] - Central/peripheral nervous system [Concussion (with or without loss of consciousness); Nerve injury] - Other [Dental injuries; Other injuries] |
| Injury incidence | Number of injuries per 1000 player hours ((Σ injuries/Σ exposure hours) ×1000). |
|  |  |
