# Supplementary material for: Injury incidence, characteristics and burden among female sub-elite futsal players: a prospective study with three-year follow-up
Source: PeerJ. 2019 Nov 5;7:e7989. doi: 10.7717/peerj.7989 (PMC6839517; doi:10.7717/peerj.7989)
Supplement: Supplemental Information 3 [file peerj-07-7989-s003.docx]

**Appendix A3: Description of the 8 criteria designed to assess risk of bias of external validity quality in the studiesT. This instrument is an adapted version of the Newcastle Ottawa Scale (NOS) for cohort studies.**

| **Criterion** | **Description of criteria** |  |
| --- | --- | --- |
| 1. Description or type of football players. | There are several types of football players (amateur vs. professional, males vs. females). Without the description regarding to the type of football players it is impossible to conclude which population the incidence rates refer to. Studies that reported a description of the football players or informed the type of football players receive a star for this criterion. Studies conducted in football tournaments (which may determine the type of football players; e.g., World cup tournaments) and which describe the race characteristics receive a star for this criterion as well. Studies that did not describe the characteristics or the type of football players, and studies conducted in football tournaments that did not describe the characteristics of the tournament did not receive a star for this criterion. | ✓ |
| 1. Definition of football-related injury. | Studies that aimed to investigate football-related injuries should present a definition of an injury informing what was considered as an injury in the study. Studies that present a definition of time-loss injury received a star for this criterion. | ✓ |
| 1. Representativeness of the exposed cohort. | (a) Truly representative of the average football players in the community*; (b) somewhat representative of the average football players in the community*; (c) selected group of users; (d) no description of the derivation of the cohort. | ✓ |
| 1. Ascertainment of exposure. | (a) Secure record*; (b) structured interview*; (c) written self-report; (d) no description | ✓ |
| 1. Demonstration that outcome of interest was not present at start of study. | (a) Yes*; (b) no. Studies that described that all football players included were injury-free at baseline received a star for this criterion. | ✓ |
| 1. Assessment of outcome. | (a) Independent blind assessment*; (b) record linkage*; (c) self-report; (d) no description. | ✓ |
| 1. Was follow-up long enough for outcomes to occur risk factors. | (a) Yes*; (b) no. Studies that carried out a follow-up period of at least 12 weeks received a star for this criterion. | ✓ |
| 1. Adequacy of follow-up of cohorts | (a) Complete follow-up of all subjects accounted for*; (b) subjects lost to follow-up unlikely to introduce bias (up to 20 % loss) or description provided of those lost*; (c) follow-up rate <80% and no description of those lost; (d) no statement. A loss to follow-up greater than 20 % may increase the risk of bias in prospective studies (Fewtrell et al., 2008). | ✓ |

T: The articles could be awarded a maximum of one star for each item. A total of 8 stars could be given for the articles.

* Articles with this alternative received a star for this criterion.
